# Microencapsulation Preservation of the Stability and Efficacy of Citrus Grandis Oil-Based Repellent Formulation against *Aedes aegypti* during Storage

**DOI:** 10.3390/molecules26123599

**Published:** 2021-06-11

**Authors:** Norashiqin Misni, Zurainee Mohamed Nor, Rohani Ahmad, Nur Raihana Ithnin, Ngah Zasmy Unyah

**Affiliations:** 1 Department of Medical Microbiology, Faculty of Medicine and Health Sciences, Universiti Putra Malaysia, Serdang 43400, Selangor, Malaysia; raihana@upm.edu.my (N.R.I.); ngah@upm.edu.my (N.Z.U.); 2 Department of Parasitology, Faculty of Medicine, University Malaya, Kuala Lumpur 50603, Malaysia; zuraineemn@um.edu.my; 3 Medical Entomology Division, Institute for Medical Research, Kuala Lumpur 50588, Malaysia; rohania@imr.gov.my

**Keywords:** stability, microencapsulation, repellent, *Citrus grandis*, *Aedes* mosquito

## Abstract

Essential oils have been widely used as an active ingredient in mosquito repellent products. However, essential oils are highly unstable and prone to degradation when exposed to the environment during storage. Microencapsulation techniques help to maintain the stability of molecules in essential oils that are sensitive to environmental stress, and therefore improve shelf life. In this study, the physical stability and efficacy of a repellent formulation consisting of encapsulated *Citrus grandis* essential oil (CGEO) were evaluated under different storage conditions over a 12-month period by comparing the formulation with a non-encapsulated formulation. The formulations were both stored under two different storage conditions, i.e., 25 ± 2 °C/60% ± 5% relative humidity (RH) and 40 ± 2 °C/75% RH ± 5%, for 12 months. Droplet size, zeta potential, and pH value were measured after 1, 6, and 12 months of storage to determine their stability. For the study of efficacy, each formulation was tested against *Aedes aegypti* under laboratory conditions. We found that the microencapsulated formulation’s physical characteristics showed insignificant changes as compared with the non-encapsulated formulation during storage. The microencapsulated formulation demonstrated better repellent effects, sustaining high protection (>80%) for 4 more hours of exposure after 12 months of storage as compared with the non-encapsulated formulation that demonstrated high protection for only an hour post application. Microencapsulation helped to preserve the stability of the formulation, which resulted in high protection being maintained for over 12 months of storage.

## 1. Introduction

Essential oils are the secondary metabolites produced and stored in the secretory glands of aromatic plants [1,2]. Independently, they play an important role in a plant’s defensive mechanisms against fungi, pathogenic microorganisms, herbivores, and insects [3]. Due to these functions, essential oils are widely used in the medicinal, pharmaceutical, cosmetics, as well as pesticide and insecticide industries [4]. In the insecticide industry, in particular, plant extracts are extensively used as active ingredients in repellent products, which are now receiving more attention from consumers than the synthetic repellents. An example of a synthetic repellent is diethyl-m-toluamide (DEET), which has been found to cause skin irritation and sensitization for some individuals and poses a toxicity effect in infants and young children. Therefore, nowadays, customers are keen to choose plant-based repellents to protect themselves against mosquito bites [5].

For decades, thousands of plants have been screened as potential sources of repellent and insecticides. However, only a few plants have been registered by the U.S. Environmental Protection Agency (U.S. EPA). Citronella, lemon, and eucalyptus oils are among the natural ingredients that have obtained approval due to their relatively low toxicity and acceptable efficacy, and therefore are often selected by many manufacturers as the ingredients in their plant-based repellents [5]. Currently, there are several plant-based repellent products available on the market that consist of the abovementioned ingredients. Unfortunately, although these products are preferred over synthetic products, the majority of them have been shown to have a short protection time of less than two hours as compared with synthetic repellents that mostly possess up to four hours of protection [6]. This attribute may be associated with the highly volatile and lipophilic constituents of the botanical extract, which are significantly subjected to conversion and degradation reactions, such as the oxidative process [7]. Furthermore, exposure to environmental factors such as air, light, and elevated temperature can cause these substances to become unstable, thus, resulting in a reduction in the quality of certain properties [8]. In addition, the breakdown of botanical insecticides is significantly faster when they are exposed to the environment as compared with synthetic insecticides [9].

Botanical insecticides, therefore, must be rigorously improved to achieve an acceptable level of effectiveness if they are to compete against synthetic insecticides [10,11]. Several techniques and methods in the formulation process have been developed in recent years to improve the stability of botanical extracts and essential oils. Microencapsulation, in particular, is one of the techniques used to protect the essential oils that can simply undergo degradation [9]. This technique involves the production of an outer coating (i.e., wall) that surrounds the particles of an essential oil (i.e., core) by using a polymer (i.e., synthetic or natural). The wall protects the unstable core material from the environment and can further control its volatility, subsequently extending the release rate of the essential oil and improving product effectiveness [12].

The preparation technique used is not the only factor that influences the effectiveness of a repellent product; the stability of its formulation is also an important component that determines the effectiveness of a product [13]. Therefore, identifying a preparation technique that improves or maintains the stability of a formulation during storage is an important objective of among studies. During the development of a repellent product, it is necessary to determine suitable storage conditions that can preserve the product’s obtained stability. For example, an unsuitable temperature can change the product’s physical stability and lead to a destabilization effect on the formulation, which directly affects the product’s quality, efficacy, and safety [14,15]. Moreover, the droplet size, polydispersity index, pH, and viscosity are among the important characteristics for determining the stability of the formulation. Accordingly, different preparation techniques produce varying characteristics.

*Citrus grandis* (L.) Osbeck, commonly known as pomelo or ”Limau Bali” is one of the species in the family of Zingiberaceae, which is widely distributed throughout the tropics, particularly in Southeast Asia. It is round in shape, greenish in color, 15–25 cm in diameter, and usually weighs about 1–2 kg [16]. The Department of Agriculture of Malaysia reported that pomelo production in Malaysia increased significantly from 1960 to 2014. Approximately 1009 ha of land had been used for pomelo plantations that produced 11,830.6 metric tons per year. Pomelo is usually consumed fresh or used for flavoring by the food and beverage industries. Meanwhile, their peel is discarded as waste. Since 30% of pomelo weight is derived from its peel, this reflects around 1956 metric tons (9.4 billion Ringgit Malaysia/USD 2.4 million) of pomelo peel being discarded per year in 2014 [17,18], without recognizing their possible nutritional and therapeutic values. Pomelo peel is composed of various bioactive compounds that several studies have proven have an antioxidant effect, and therefore are beneficial for food and pharmaceutical companies [16]. The oils from citrus cultivars such as *Citrus grandis, Citrus sinensis, Citrus paradisi, Citrus reticulata, Citrus limon, and Citrus aurantium* have been shown to have strong activity against *Aedes albopictus* under laboratory conditions [19,20,21,22]. These oils have secondary metabolites, which help plants deter insect pests [23]. Therefore, due to its insecticidal properties, it is worthwhile investigating pomelo peel for managing vector mosquitoes.

In this study, pomelo peel oil was selected and subjected to a microencapsulation technique in order to produce a microencapsulated *Citrus grandis* essential oil (CGEO)-based repellent. The stability and efficacy testing for different storage conditions, i.e., short (1 month), accelerated (6 months), and long term (12 months) were conducted accordingly, and then compared to a non-encapsulated CGEO-based repellent formulation.

## 2. Results

### 2.1. Organoleptic Characteristics of the Formulations

The suspension of semisolid microencapsulated CGEO presented as a milky white liquid with no essential oil observed either on the surface of the suspension or after centrifugation of the discarded aqueous supernatant. The microencapsulated and non-encapsulated formulations both presented a white color appearance, specific odors, light texture, good homogenization, and easy spreadability when applied on the skin right after their preparation.

### 2.2. Stability Characteristics of the Formulations

#### 2.2.1. Centrifugation Test

Under the short-term, accelerated, and long-term storage conditions, the microencapsulated and non-encapsulated formulations both presented no phase separation when stored at 25 °C. In contrast, under the 40 °C storage condition, both presented phase separation after 6 and 12 months of storage.

#### 2.2.2. Organoleptic Test

In the organoleptic study, it was observed that all formulations showed no change in appearance, color, smell, and texture during the short-term, accelerated, or long-term storage when stored under 25 °C storage conditions. Sediment, crystallization, and phase separation were also absent (data not shown). Upon skin application, no residue was detected and an insignificant change in spreadability was observed during these periods. However, under the 40 °C storage condition, both formulations showed a slight change in their color (i.e., yellowish), produced a strong odor, presented phase separation, and became very light in texture after 6 months of storage. Furthermore, 12 months of storage caused the formulations to turn slightly darker in color and to release an irritating odor. Phase separation was further observed, a change in spreadability (very light) was noted, and an oily feeling upon application on the skin was also detected.

#### 2.2.3. Particle Size and Zeta Potential

**Figure 1:**
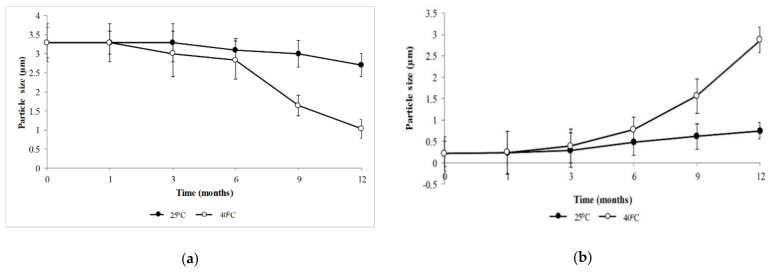
Particle sizes of the formulations. (**a**) The microencapsulated formulation; (**b**) the non-encapsulated formulation. Stored at 25 ± 2 °C/60% ± 5% RH and 40 ± 2 °C/75% ± 5% RH. The results are represented by mean ± SD.

Right after its preparation, an average particle size (Z-average) of the microencapsulated formulation was 3.29 µm, while the non-encapsulated formulation was 0.21 µm. Figure 1 shows the changes in particle size for the microencapsulated formulation (Figure 1a) and non-encapsulated formulation (Figure 1b) during the study period in different storage conditions. Under the 25 °C storage condition, the average particle size of the microencapsulated formulation showed stability during the first three months. However, after 3 to 12 months of storage, particle sizes were reduced by 17.9%, whereas under the 40 °C storage condition, particle sizes showed a 68.7% reduction after 1 month of storage. In contrast, the non-encapsulated formulation average particle size showed an opposite trend, i.e., 72% and 92.7% reduction when stored at 25 °C and 40 °C storage conditions, respectively.

**Figure 2:**
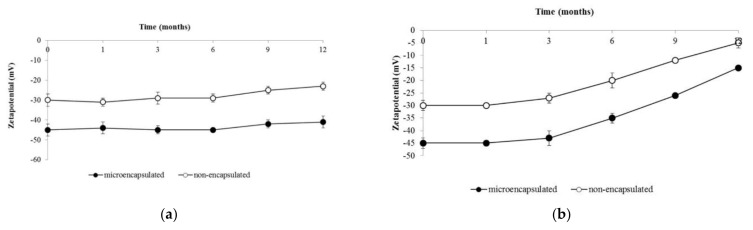
Zeta potential values of the microencapsulated and non-encapsulated formulations. (**a**) Stored at 25 ± 2 °C/60% ± 5% RH; (**b**) stored at 40 ± 2 °C/75% ± 5% RH. The results are represented as mean ± SD.

Figure 2 shows the zeta potential variation of both formulations stored at 25 °C (Figure 2a) and 40 °C (Figure 2b). Right after the preparation, the microencapsulated formulation demonstrated high stability (−45 mV), while the non-encapsulated formulation demonstrated moderate stability (−30 mV). Under the 25 °C storage condition, both formulations showed no significant changes in the average zeta potential values during the short and accelerated terms of storage (*p* < 0.05). However, after 6 months of storage, the microencapsulated formulation showed a 9% reduction in the average zeta potential value, while the non-encapsulated formulation showed a 20% reduction. Moreover, after 12 months of storage, the microencapsulated formulation presented high stability (−41 mV), whereas the non-encapsulated formulation demonstrated a slight dispersion (−23 mV) in their emulsion system (Figure 2a). Under the 40 °C storage condition, the microencapsulated formulation demonstrated agglomeration (−15 mV), while the non-encapsulated formulation indicated precipitation and phase separation (−5 mV) in the emulsion system after 12 months of storage (Figure 2b).

#### 2.2.4. pH Measurement

**Figure 3:**
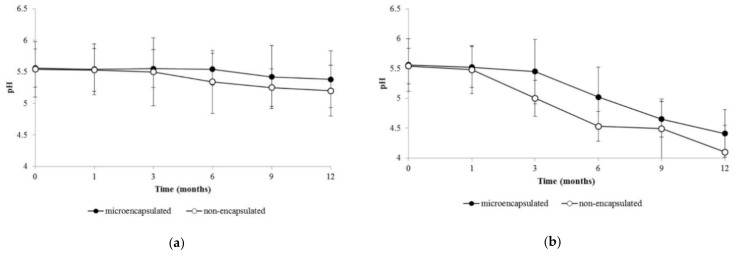
pH measurements of the microencapsulated and non-encapsulated formulations. (**a**) Stored at 25 ± 2 °C/60% ± 5% RH; (**b**) stored at 40 ± 2 °C/75% ± 5% RH. The results are represented as mean ± SD.

In this study, both formulations were prepared to have a pH of 5.0 to 5.5 and the pH measurements were done right after the preparations. In Figure 3, it can be observed that, under the 25 °C storage condition, the pH values decrease significantly from 5.56 to 5.38 and from 5.54 to 5.20 for the microencapsulated and non-encapsulated formulations, respectively (*p* < 0.05) (Figure 3a). Under the 40 °C storage condition, a reduction in pH values was observed significantly after 12 months of storage for both formulations (Figure 3b) in which all values were below 5.0 (*p* < 0.05).

### 2.3. Efficacy Study

**Figure 4:**
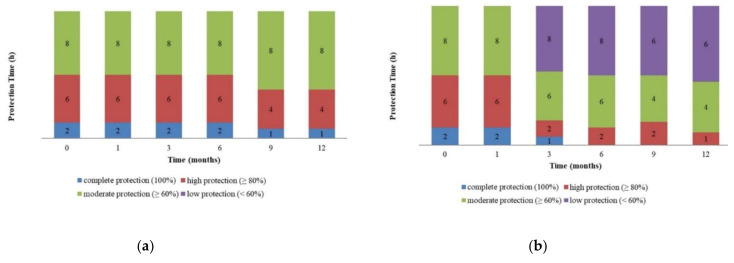
Repellent effects. (**a**) Microencapsulated formulation; (**b**) non-encapsulated formulation. Stored at 25 ± 2 °C/60% ± 5% RH and 40 ± 2 °C/75% ± 5% RH.

According to the stability study, the 25 °C storage condition was observed to be the most suitable condition for maintaining the stability of the formulations. Therefore, the formulations stored under this condition were tested for their repellent effects to ensure the effectiveness during storage. The trends of protection time over 12 months for microencapsulated and non-encapsulated formulations are shown in Figure 4. Both formulations showed similar efficacy levels right after the preparation, i.e., providing up to 2 h of complete protection (100%), >80% at 6 h, and >60% at 8 h post application, accordingly. After 6 months of storage, the microencapsulated formulation was able to maintain this efficacy level (Figure 4a), while the non-encapsulated formulation yielded a reduction in protection time after 3 months of storage. For the latter formulation, a complete protection time was observed at 1 h, high protection at 2 h, and moderate protection at 8 h post application (Figure 4b). After 9 months of storage, the microencapsulated formulation showed complete protection for 1 h post application (Figure 4a). In contrast, the non-encapsulated formulation showed no complete protection even after 6 months of storage (Figure 4b).

## 3. Discussion

Recently, there are many topical mosquito repellents on the market that are available in many forms, i.e., sprays, creams, lotions, aerosols, oils, evaporators, patches, and canisters. Despite the remarkable advancement in insect repellent research, the menace of vector-borne diseases is yet to be effectively controlled and the main reason for this failure is poor user compliance with respect to using the marketed repellents [6]. For example, topical repellents need to be reapplied frequently, which results in an inconsistent amenability and inaccurate application. Thus, they are considered to be unreliable vector control tools [24]. Therefore, the design and development of topical repellent formulations, is an approach that can be explored to overcome this problem and enhance user compliance and acceptability.

In this study, a plant-based essential oil was successfully encapsulated by using an interfacial precipitation technique as described in the literature [25,26,27]. As such, no phase separation was shown by the formulation right after its preparation, indicating effective homogenization of the aqueous and oil phases and successful emulsification [28]. Furthermore, the organoleptic characteristics suggested that both formulations fulfilled the criteria in sensorial quality, which included a non-greasy feeling, light texture, spreadability, and pleasant feeling and aroma. Such sensorial qualities also play an important role in determining consumer compliance. Cheng et al. (2009) [28], in their study, reported that most consumers express dislike for a product that is not smooth and emits a greasy feeling.

During storage, environmental factors such as temperature and humidity can lead to destabilization of a formulation’s physical characteristics, subsequently affecting its efficacy and safety [14]. Therefore, suitable storage conditions are crucial to minimize environmental effects on the formulation. However, another factor to consider when determining formulation stability is, namely, the type of formulation [28]. This study suggested that different formulations demonstrated different physical stabilities during storage.

A centrifugation test can provide fast information regarding the emulsion stability properties of a formulation. An emulsion is unstable when phase separation occurs after centrifugation [29]. In the present study, we demonstrated that throughout 12 months of storage, both formulations presented no phase separation when stored under the 25 °C storage condition as compared with the 40 °C storage condition, indicating that stability of the formulation was maintained under the 25 °C storage condition Moreover, the organoleptic assays demonstrated that, under the 25 °C storage condition, both formulations presented no changes in their physical appearance over 12 months of storage. However, under the 40 °C storage condition, both formulations demonstrated changes in terms of their physical appearance after six months of storage. This was consistent with the centrifugation study outcomes, whereby the physical instability of the emulsion system was observed after six months of storage when stored under the 40 °C storage condition. Therefore, a reduction in the physical stability of the emulsion system causes phase separation and can also lead to changes in the appearance, consistency, spreadability, and performance of a formulation [30].

Moreover, this study demonstrated that, under the 25 °C storage condition, the microencapsulated formulation presented no significant reduction in particle size over 12 months of storage (*p* > 0.05). The non-encapsulated formulation, however, presented a significant upsurge in particle size after 3 months of storage (*p* < 0.05), suggesting that encapsulation helped to retain particle size when stored under the 25 °C storage condition. In addition, this study revealed that both formulations presented contrary trends in particle size during the 12 months of storage. This finding parallels a previous study that reported that the particle size of microencapsulated DEET showed a declining trend after 3 months of storage [26]. The reduction was attributed to the release of DEET within the microcapsule into the continuous phase, thus decreasing particle size [26]. A similar mechanism may be involved for microencapsulated formulation in this study. Contrastingly, the non-encapsulated formulation showed an increase in particle size indicative of coalescence between the particles. This finding is similar to a previous study that found an increased particle size during the study period due to the coalescence phenomenon in a non-encapsulated formulation [31,32]. Accordingly, exposure to higher temperature can cause an exaggeration of these mechanisms, which, if it continues, the dispersed phase separates and the emulsion breaks [31]. In this study, under the 40 °C storage condition, both formulations showed significant changes in their particle sizes while the phase separation was observed at the end of the study period.

Similarly, the zeta potential measurement provides the charge on a particle surface. On the one hand, high values, whether negative or positive, lead to repulsive forces between particles, which improve the repellency between particles, thus inhibiting aggregations [33]. On the other hand, low potential values indicate the absence of repulsive interaction and result in particle aggregation and instability of the emulsion system [33]. Right after the preparation, the microencapsulated formulation demonstrated high stability (−45 mV), while a moderate stability (−30 mV) was seen for the non-encapsulated formulation. During storage under the 25 °C condition, the microencapsulated formulation showed no significant changes in its zeta potential value over 12 months of storage, while the non-encapsulated formulation presented a significant reduction after 6 months of storage. These results are in line with the theory regarding zeta potential value, i.e., in the case of microencapsulated formulations, high zeta potential values provide strong repulsive forces between particles, preventing aggregations and allowing the emulsion system to be stable. On the contrary, non-encapsulated formulations with low zeta potential values cause low repulsive forces and lead to particle aggregation and instability of the emulsion system [33].

Under the 40 °C storage condition, 6 and 12 months of storage resulted in instability of both formulations, i.e., the microencapsulated formulation demonstrated agglomeration, whereas the non-encapsulated formulation indicated precipitation and phase separation occurred. Temperature is one of the factors that can affect zeta potential values. Here, exposure to high temperatures decreased the repulsive forces between particles, which then encouraged aggregation and caused the emulsion system to become unstable [32,34]. Other than temperature, the pH of an emulsion also contributes to changes in the zeta potential value, i.e., the zeta potential value decreases with a reduction in pH. The results obtained from the pH assay in this study were consistent with this. As mentioned in previous studies, a reduction in zeta potential charge on the particles can be observed when the pH value is decreased [35].

Alterations in pH values can cause a formulation to become incompatible with the skin and may cause skin irritation [29]; therefore, it is important to detect any pH alterations during storage. The results from the stability study indicated that although the pH was shown to reduce for both formulations over 12 months of storage under the 25 °C storage condition, the change was within the range of pH values of 5.0 to 5.5. Therefore, this range is still suitable for topical application [36]. Regardless, the decreasing pH values in both formulations may be due to the presence of free essential oil in the emulsion system following essential oil release from the microcapsule in terms of the microencapsulated formulation. Otherwise, it may be due to the particle breakdown in the non-encapsulated formulation. Under the 40 °C storage condition, the pH value was below 5.0 (acidic state), and therefore was likely unsuitable for topical application.

The data regarding the repellent activity showed that both formulations demonstrated a reduction in such activity throughout the study period. However, the microencapsulated formulation was considered to be better than the non-encapsulated formulation as it possessed repellent activity that was maintained up to 6 months of storage as compared with the non-encapsulated formulation’s 3 months of storage. The superior repellent activity is possibly due to the wall surrounding the particle, which allows the slow release of essential oil into the environment upon application of the formulation on the skin. Microencapsulation also yields protection for EOs against oxidation when exposed to the environment and chemical interactions with other chemicals inside the formulation, thereby improving their functional activity as a repellent. Accordingly, these features also help to maintain the emulsion stability of the formulation, which is consistent with the results obtained from stability testing. The testing demonstrated that the microencapsulated formulation showed better stability as compared with the non-encapsulated formulation across a similar storage condition and time of storage.

## 4. Materials and Methods

### 4.1. Chemical Reagents

Cetyl alcohol, stearic acid, vanillin, Span 80, Tween 60, carboxymethyl cellulose (CMC), polyethylene glycol (PEG) 3350, benzalkonium chloride (BKC), and diethyl-m-toluamide (DEET) were purchased from Sigma-Aldrich, Saint Louis, USA. Dow corning 200 was purchased from Dow Corning, Michigan, USA. Meanwhile, jojoba oil, sweet almond oil, coconut oil, emulsifying wax, shea butter, and cocoa butter were purchased from BF1 Malaysia, Selangor, Malaysia. The mosquitoes as subjects in the repellent efficacy study were provided by the Institute for Medical Research of Malaysia (IMR). Susceptible strains and nulliparous three- to seven-day-old adults were used as test species.

### 4.2. Essential Oil Extraction

The fruit of *Citrus grandis* (CG) were purchased from a wholesale market in Selangor, Malaysia and used as received to maintain their freshness. The peel was cut into small pieces before subjected to hydrodistillation in a Clevenger-type apparatus for 6 h to obtain the essential oil (CGEO) [37]. The extracted oil was dried over anhydrous magnesium sulfate to dry the oil before being stored in an airtight bottle and kept at 4 °C for further use.

### 4.3. Encapsulation Procedure

CGEO was encapsulated by using interfacial precipitation chemistry to form a polysaccharide film around the dispersal droplet [25,26,27]. Briefly, the microcapsule walls were formed in two steps by reacting to the amphiphilic macromolecule CMC with a complementary reactant BKC. The first step was the formation of emulsions containing droplets of core material (i.e., CGEO) in the first wall-forming reactant solution (i.e., CMC) that preferentially accumulated at the droplet surface by polar solvent forces. The second step was the addition of the second wall forming reactant (i.e., BKC) to the system and spontaneously precipitating the CMC to form a membrane-like wall surrounding each droplet.

For this study, the microencapsulation of CGEO involved several phases as follows: Phase A was the amalgamation of CGEO with an adjuvant to form a core phase consisting of 60% active ingredient and 40% adjuvant. Dow Corning 200 (silicon oil) and vanillin were mixed and blended in a 200 mL beaker using a magnetic stirrer bar set to rotate at a minimal speed of 200 rpm at 60 °C. Then, the solution was allowed to cool to 45 °C before the active ingredient was added to the mixture. Stirring was continued to complete the process. In another beaker, all ingredients for Phase B (i.e., Cetyl alcohol, PEG 3350, Span 80, and Tween 60) were heated to melting point (60 °C) and stirred to mix completely. Then, the Phase A mixture was slowly added into the Phase B mixture and stirring was continued to form an emulsion, thereby referred to as the core emulsion mixture.

**Figure 5:**
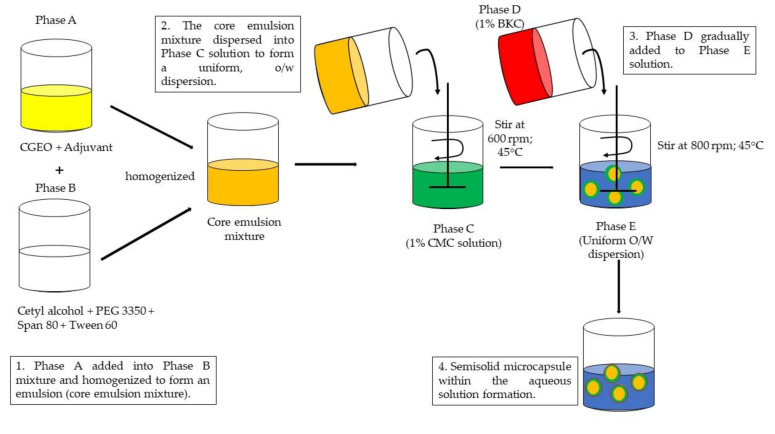
Schematic representation of the microencapsulation process of the *Citrus grandis* essential oil (CGEO).

Phase C was the aqueous solution of the first wall-forming reactant, which was combined with distilled water to make a 1% solution and mixed using a 40 mm diameter four-bladed propeller at 600 rpm and 45 °C. Then, the core emulsion mixture was dispersed into the Phase C solution at the same rotation speed and temperature. Stirring was maintained for 1 h to produce a uniform oil-in-water dispersion (Phase E). Meanwhile, the Phase D mixture consisted of the second wall-forming reactant (i.e., BKC) and distilled water, which was gradually added to the Phase E emulsion. Concurrently, the stirrer speed was slowly increased to 800 rpm in order to achieve the formation of a microcapsule wall. Then, this mixture was removed from heating after 120 s and allowed to cool to ambient temperature by stirring to complete the reaction, resulting in a semisolid microcapsule within the aqueous solution. Next, the resulting mixture was transferred into a container with a secured screw cap after an ambient temperature was achieved. In summary, Figure 5 shows the schematic representation of the microencapsulation process of CGEO and Table 1 shows the amount of each ingredient added during the encapsulation process.

### 4.4. Formulation into a Lotion Base

The microencapsulated CGEO was formulated into a lotion base to produce a cosmetic repellent product. Emulsifying wax NF, stearic acid, shea butter, cocoa butter, coconut oil, sweet almond oil, and jojoba oil were mixed using a propeller at 400 rpm and heated at 70 °C. Then, the mixture was allowed to cool to 45 °C before glycerine, aloe vera gel, and a microencapsulated CGEO were added, while continuously stirring until a uniform mixture was produced. For the non-encapsulated formulation, a similar procedure was done in which 20% of CGEO oil was added into the mixture to replace the microencapsulated CGEO.

### 4.5. Physical Stability Testing of the Formulation

Stability testing was carried out to evaluate the formulation stability under the influence of various environmental factors with time. The storage conditions and testing frequency of the final products were performed by following the WHO guidelines for stability study of pharmaceutical products [38]. The microencapsulated and non-encapsulated formulations were packaged accordingly in impermeable polypropylene containers and stored under different conditions, namely controlled room temperature (25 ± 2 °C/60% ± 5% RH) and 40 ± 2 °C/75 ± 5% RH. After 1 month (short-term), 6 months (accelerated term), and 12 months (long-term) of storage, these formulations were evaluated for their stability.

### 4.6. Centrifugation Assay

This test was carried out by weighing 2 g of each sample, followed by centrifugation at 3000 rpm for 30 min for phase separation [29].

### 4.7. Organoleptic Assay

The organoleptic features of the samples were examined via the changes in color, smell, texture, sediment, phase separation, crystallization, absence/presence of residue when applied on skin, and hardness of the lotions [29].

### 4.8. Particle Size and Zeta Potential Analyses

First, 20 mg of samples were dispersed in 50 mL of deionized water and sonicated at 25 °C for 15 min. Then, the solutions were transferred into a folded capillary cell (i.e., polycarbonate with gold-plated electrodes) to test for the particle size and zeta potential using a Zetasizer Nano (Malvern Instrument Ltd., Worcestershire, the United Kingdom) at 25 °C. The mean particle diameter was calculated using the differential size distribution processor (SDP) intensity analysis program. The zeta potential values were graded according to the Morrison and Ross 2002 [33] guideline, i.e., values from ±60 to ±40 mV indicate high stability, ±40 to ±30 mV indicate moderate stability, ±30 to ±15 mV indicate light dispersion, and ±15 to ±10 mV indicate agglomeration.

### 4.9. pH Measurement

Here, 1 g of sample was diluted in distilled water measuring up to 10 mL, which was, then, homogenized before the pH measurement was carried out by using a pH meter (Mettler Toledo, Greifensee, Switzerland) [29].

### 4.10. Efficacy Testing

Evaluation of repellent activity for each formulation followed the methods provided by the Malaysian Standard Method for repellent MS 1497:2000 [39], which were modified from the World Health Organization (WHO) guidelines [40]. Bioassays were conducted using a 60 × 60 × 60 cm screened cage with two 15 cm diameter circular openings fitted with cloth sleeves. The cage had two compartments separated by a clear acrylic plastic in the middle. Nulliparous three- to seven-day-old adult mosquitoes were used in the bioassays.

A fresh batch of 25 female *A. aegypti* mosquitoes was introduced into each compartment through the circular opening. Two 25 cm^2^ areas were drawn on top of the hands of the human volunteers. One area was untreated (control) while the other was pretreated with 0.4 g of a test formulation. Before treatment, both hands were covered with rubber gloves that had a 25 cm^2^ opening up to the wrist level to confine the mosquito bites to the exposed areas only. Both hands were exposed simultaneously in a cage for three min and the number of mosquito bites was recorded. The assessment periods were set at 1, 2, 4, 6, and 8 h post application. The effectiveness of the formulations was based on the percent reduction in mosquito bites on the treated arms as compared with the untreated arms (control) using the following formula:

% protection reduction = [(C − T)/C] × 100,(1)

where *C* is the total number of mosquito bites on the control and *T* is the total number of mosquito bites on the treated arms. Each lotion formulation was tested on five human volunteers for three iterations.

### 4.11. Statistical Analysis

The stability and efficacy studies were done in triplicate and the mean value, standard deviation (SD), and standard error mean (SEM) of each result was obtained using a descriptive analysis via the use of SPSS version 21.0 software. The mean values were analyzed using parametric analysis via two-way mix-split ANOVA design (SPANOVA) to compare the mean differences between each formulation with the level of significance at *p* < 0.05.

## 5. Conclusions

Marked encapsulation application in plant-based repellent product development helped to improve the stability and efficacy of the formulation, which were successfully observed in this study. Although both formulations presented stability when stored under 25 ± 2 °C/60% ± 5% RH condition, the microencapsulated formulation appeared to demonstrate an excellent ability to retain its physical stability and efficacy over 12 months of storage as compared with the non-encapsulated formulation. However, the microencapsulated formulation also seemed to share similar stability characteristics with the non-encapsulated formulation under the 40 ± 2 °C/70% ± 5% RH storage condition, whereby both formulations presented instability in their respective emulsion systems. Therefore, this suggests that a microencapsulation formulation cannot maintain its physical stability against high temperature, especially when continuously exposed over a long period of time. More investigation on the improvement of the formulation technique for examples by using different encapsulation materials or a higher ratio of CMC is necessary to improve the stability at high temperature.

## Figures and Tables

**Table 1:** Composition of the encapsulation process.

Ingredients	Mass (g)
Phase A	
CGEO	60
Dow Corning 200	20
Vanillin	20
Phase B	
Cetyl alcohol	30
PEG 3350	10
Span 80	5
Tween 60	5
Phase C	
1% CMC solution	20
Distilled water	~100
Phase D	
1% BKC solution	10

## Data Availability

Data is contained within the article.

## Sample Availability

Samples of the compounds are available from the authors.
